# Lycium barbarum Polysaccharides Promote Maturity of Murine Dendritic Cells through Toll-Like Receptor 4-Erk1/2-Blimp1 Signaling Pathway

**DOI:** 10.1155/2020/1751793

**Published:** 2020-12-01

**Authors:** Xiangguo Duan, Yaru Lan, Xiaoyu Zhang, Shaozhang Hou, Jian Chen, Bin Ma, Yuhan Xia, Chunxia Su

**Affiliations:** ^1^Department of Laboratory Medicine, College of Clinical Medicine, Ningxia Medical University, Yinchuan 750004, China; ^2^Department of Laboratory Surgery, General Hospital of Ningxia Medical University, Yinchuan 750004, China; ^3^Ningxia Innovation Team of the Foundation and Clinical Researches of Diabetes and Its Complications, Yinchuan 750004, China; ^4^School of Basic Medical Sciences, Ningxia Medical University, Yinchuan 750004, China; ^5^The People's Hospital of Caoxian, Heze 274400, China; ^6^Guolong Hospital, Yinchuan 750004, China; ^7^Department of Oncology Surgery, The First People's Hospital of Yinchuan, Yinchuan 750004, China; ^8^General Hospital of Ningxia Medical University, Yinchuan 750004, China

## Abstract

In previous studies, Lycium barbarum polysaccharides (LBP), a traditional Chinese medicine, can promote immature dendritic cells (DCs) to mature. However, the molecular mechanisms by which LBP works are not yet elucidated. Here, we found that LBP can induce DCs maturation, which is mainly characterized by the upregulation of MHCII and costimulatory molecules (CD80, CD86), and increase the production of IL-6 and IL-4. Furthermore, we found that LBP could increase the mRNA and protein expression of TLR4, p38, Erk1/2, JNK, and Blimp1 signal molecules. More interestingly, after blocking by Toll-like receptor 4 inhibitor, Resatorvid (TAK 242), the mRNA and protein expression of TLR4, Erk1/2, and Blimp1 was significantly decreased while the expression of p38 and JNK has not changed. Then, we found that after blocking by p38 inhibitor (SB203580), Erk inhibitor (PD98059), and JNK inhibitor (SP603580) separately, Blimp1 protein expression was significantly reduced; after downregulating Blimp1 by Blimp1-siRNA, the production of IL-6 was reduced. In conclusion, our results indicate that LBP can induce maturation of DCs through the TLR4-Erk1/2-Blimp1 signal pathway instead of the JNK/p38-Blimp1 pathway. Our findings may provide a novel evidence for understanding the molecular mechanisms of LBP on activating murine DCs.

## 1. Introduction

Chinese herbal medicine has a long history used to treat many kinds of human diseases. Lycium barbarum polysaccharides (LBP) are the major biological active ingredient of Lanthanum which consist of six kinds of monosaccharides such as arabinose, glucose, galactose, mannose, xylose, and rhamnose and have a variety of biological and pharmacological functions, including antioxidant, anticancer and antiradiation activities [1–4]. From immunology aspect, what we understood about LBP is that it mainly regulated the functions of T cells, CTL cells, NK cells, peritoneal macrophages, and partially dendritic cells (DCs). Some studies have shown that LBP can increase the expression of surface molecular CD11c and MHCII and the secretion of IL-12p40 in mouse bone marrow-derived dendritic cells (BMDCs) [5]. However, the molecular mechanisms by which LBP works are not yet elucidated.

DCs are the most powerful antigen presenting cells (APC) known to act as a bridge between innate and adaptive immune responses [6]. Immature DCs have a strong ability to swallow antigens, while mature DCs have a strong capacity to present antigens. A variety of stimulus can mature DCs by binding to its cell surface receptors, which are characterized by high expression of CD40, CD80, CD86, and MHCII molecules [7]. Mature DCs are important modulators of immune response, and they are the key modulator in inducing several CD4^+^ effector T cell subsets by high secretion of cytokines. DCs can provide T cell signals as antigenic peptide, costimulatory molecules, and cytokines, thereby activate the initial T cell differentiation, and participate in the immune response [8, 9]. IL-6 is a key cytokine secreted by mature DCs that can effectively induce differentiation of T follicular helper (Tfh) cell [10–13]. Tfh cells are able to promote humoral immune response by helping B cells to differentiate into plasma cells, which can produce high-affinity antibodies [14–16].

Numerous studies have shown that B lymphocyte-induced mature protein 1 (Blimp1) encoded by *Prdm1* is the main molecule for B cells to differentiate into plasma cells and CD4^+^ T cells to secrete cytokines [17]. Meanwhile, Blimp1 is also involved in lipopolysaccharide- (LPS-) induced primary B lymphocyte activation, macrophage differentiation, and DC maturation [18, 19]. Although Blimp1 is rarely expressed in bone marrow-derived DCs, it still plays a crucial role in the tolerogenic function of DCs. Reducing the expression of Blimp1 in DCs can lead to abnormal activation of the adaptive immune response, which may participate into DC differentiation and maturation [20–22].

Up to now, TLR2/4 is proposed to be a possible receptor for polysaccharides. Japanese scholars first reported that safflower polysaccharide can activate the transcription factor NF*κ*B signal pathway through TLR4 [23]. Following that, a lot of research has also shown that many plant polysaccharides can activate the TLR4-MAPK signaling pathway in cells and improve the production of TNF-*α*, IL-12, and IL-1*β* [24, 25]. However, there is currently no clear evidence of whether LBP are involved in the TLR-MAPK signaling pathway. In this study, we are interested to look at how LBP works to regulate the maturation of DCs and what is the molecular mechanisms of LBP on activating murine DCs.

## 2. Materials and Methods

### 2.1. Mice

C57BL/6 mice (SCXK-(jing) 2016-0006) were purchased from the Laboratory Animal Center of Ningxia Medical University (Yinchuan, Ningxia, China). All mice were housed in the Animal Experiment Center of Ningxia Medical University. All mouse procedures were approved by the Institutional Animal Care and Use Committee of the Ningxia Medical University.

### 2.2. Materials

LBP was purchased Shanxi Ciyuan Biotechnology (No.CY190218). Fetal bovine serum was purchased from PNA, Paisley, UK. Recombinant Murine GM-CSF and recombinant Murine IL-4 were both from PeproTech (USA). Lipopolysaccharide was from Solarbio, China.

### 2.3. Generation of DCs from Bone Marrow

Immature DCs were generated from the bone marrow using a protocol slightly modified from a previous study [5]. The cells were resuspended in complete medium containing 20 ng/mL GM-CSF and 10 ng/mL IL-4. The medium was disposed of half by the next day. Nonadherent cells were collected after 7 days of cultivation. DCs were treated with various concentrations of LBP (0, 50, 100, and 200 *μ*g/mL) for 30 min. In the positive control, 10 ng/mL LPS was added. In inhibition experiments, DCs pretreated with 10 *μ*M Resatorvid (TAK 242) (MCE, USA), 2.5 *μ*M BAY 11-7082 (Ab Mole, USA), 5 *μ*M SB203580 (Ab Mole, USA), 10 *μ*M PD98059 (Ab Mole, USA), 50 *μ*M SP600125 (Ab Mole, USA) for 1 h were treated with LBP (200 *μ*g/mL) or LPS (100 ng/mL) for 30 min.

### 2.4. DCs Phenotypic Characterization by Flow Cytometry (FCM)

DCs were seeded in 6-well plates at 1 × 10^6^ per well and incubated for 24 h with LBP (0, 50, 100, and 200 *μ*g/mL) and LPS (100 ng/mL). DCs were collected and stained with PE-Cy7-conjugated CD11c, V500-conjugated MHCII, BV421-conjugated CD86, and Percp-Cy™ 5.5-conjugated CD80 (BD Bioscience, USA). Stained cells were analyzed by FCM and displayed as mean fluorescence intensity (MFI), and the higher MFI means higher expression of detected molecules.

### 2.5. Cytokine Examination Assay

On day 7, DCs were seeded in 6-well plates at 1 × 10^6^ per well and incubated for 24 h with LBP (0, 50, 100, and 200 *μ*g/mL) and LPS (100 ng/mL). DC culture supernatants were collected to test the various cytokines by a sandwich enzyme-linked immunosorbent assay (ELISA) kit (BD Bioscience, USA) according to the manufacturer's instructions.

### 2.6. Taqman-PCR Assay

On day 7, DCs were coincubated with LBP (0, 50, 100, and 200 *μ*g/mL) for 30 min, and then, the RNA was extracted using Tiangen RNA Extraction Kit and then reverse-transcribed into cDNA using 1 *μ*g RNA. The cDNA was stored at -80°C for later use. PCR was performed in a 2720 Thermal Cycler (Gene Co. Ltd.). The mRNA levels of TLR4, JNK, p38, Erk1, Erk2, and Blimp1 were normalized versus those of *β*-actin.

### 2.7. Western Blotting Assay

25 micrograms of total protein was electrophoresed by 8% SDS PAGE. The transferred membrane was blocked in 5% nonfat milk for 1.5 h, and then, the primary antibody was incubated at 4°C overnight. The secondary antibody labeled with horseradish peroxidase was detected using an ECL kit. Images were then analyzed by ImageJ 6.0 software. *β*-Actin was used as a control.

### 2.8. RNA Silencing Assay

For RNA silencing experiments, small interfering RNA of Prdm1 (siRNA) was purchased from QIAGEN (Germany) and transfected into DCs using HiPerFect Transfection Reagent (Germany). The effect of knockdown was checked by Western blot and ELISA (24 h after transfection).

### 2.9. DC Stimulation with LBP In Vivo

Both LBP (0, 10, 20, 40 mg/kg) and LPS (1 mg/kg) were administered intraperitoneally to C57BL/6 mice in three groups. After 7 days, DCs were isolated from the spleen and detected for TLR4, Erk1/2, and Blimp1 expression by Western blot.

### 2.10. Statistical Analysis

Statistical analysis was performed using Prism 5.0 (GraphPad). Measurement data were shown as the mean ± SD. Statistical significance was calculated by one-way analysis of variant: *P* < 0.05 was considered the statistical significance.

## 3. Results

### 3.1. Phenotypic and Functional Change of DCs after Treatment by LBP

**Figure 1:**
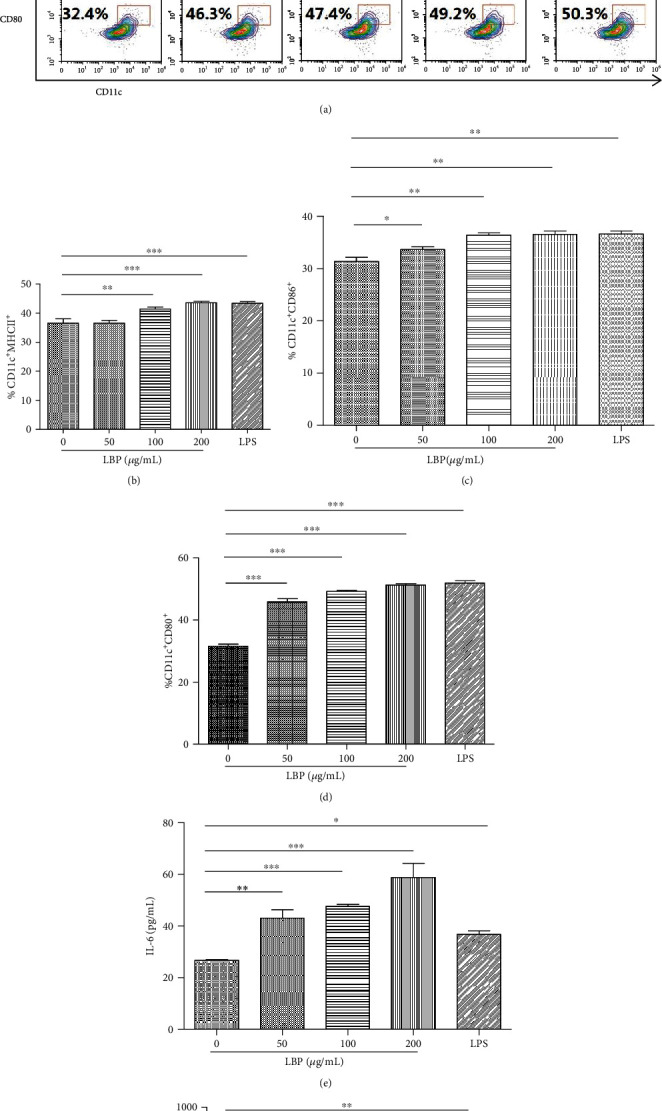
Phenotypic and functional change of DCs after treatment by LBP. After being cocultured with DCs for 24 h with LBP (0, 50, 100, 200, and 200 *μ*g/mL) and LPS (100 ng/mL), FCM detected the double positive CD11c^+^MHCII^+^ (b), CD11c^+^CD80^+^ (c), and CD11c^+^CD86^+^ (d) cells in five groups. Supernatants were measured using ELISA kits for IL-6 (e) and IL-4 (f). Values are the mean and SD of 3 independent experiments. ^∗^*P* < 0.05, ^∗∗^*P* < 0.01, and ^∗∗∗^*P* < 0.001.

To investigate the potential role of LBP in DCs maturity, we started by analyzing the changes of DCs surface molecular after LBP treatment. After 24 h poststimulation, the expression of the DC surface markers MHCII, CD86, and CD80 were detected by FCM. First, Figure 1(a) shows the percentage of CD11c^+^MHCII^+^, CD11c^+^CD86^+^, and CD11c^+^CD80^+^ cells in different experimental groups, and Figures 1(b)–1(d) show the statistical charts of MHCII-, CD86-, and CD80-positive cells. The results show that LBP can increase the percentage of mature DCs. In addition, as displayed in Figures 1(e) and 1(f), LBP significantly increases the secretion of IL-6 and IL-4 in DCs except for the LPS group. Our data suggested that LBP can promote DCs phenotype and functional maturity; thus, we next focused on analyzing the molecular mechanism of LBP on DCs.

### 3.2. LBP Improve TLR4 Expression on the Cell Surface of DCs

**Figure 2:**
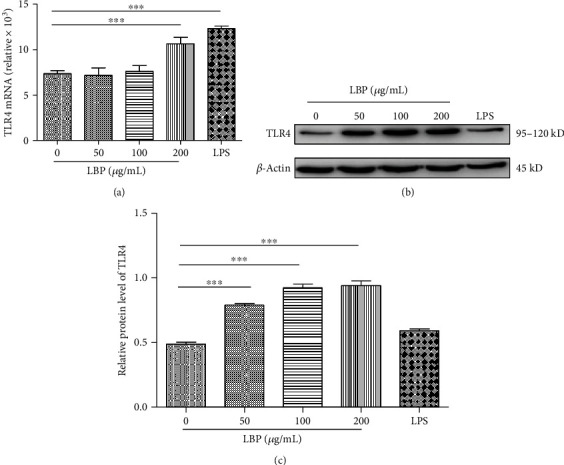
LBP improve TLR4 expression on the cell surface of DCs. DCs were stimulated for 30 min with LBP (0, 50, 100, and 200 *μ*g/mL) and LPS (100 ng/mL), respectively. After stimulation, the cell RNA and protein were extracted; gene and protein expression of TLR4 was detected by Taqman-PCR (a) and Western blot (b) and (c) separately. Values are the mean and SD of 3 independent experiments. ^∗^*P* < 0.05, ^∗∗^*P* < 0.01, and ^∗∗∗^*P* < 0.001 were considered statistically significant different from control.

Numerous reports show that increased expression of TLRs is one of the immunomodulatory mechanisms of plant polysaccharides played. Therefore, we further detected the expression of TLR4 on DCs after LBP-treated by Taqman-PCR and Western blot. As indicated in Figure 2, a high expression of TLR4 mRNA (Figure 2(a)) and protein (Figure 2(b)) was observed in DCs after stimulation for 30 min with both LPS and LBP. Furthermore, LBP also showed an increase of TLR4 mRNA and protein level in a dose-dependent manner. Our results suggested that TLR4 plays a pivotal role in regulating the LBP-treated DCs.

### 3.3. LBP Increase MAPK, Blimp1 mRNA, and Protein Expression in DCs

**Figure 3:**
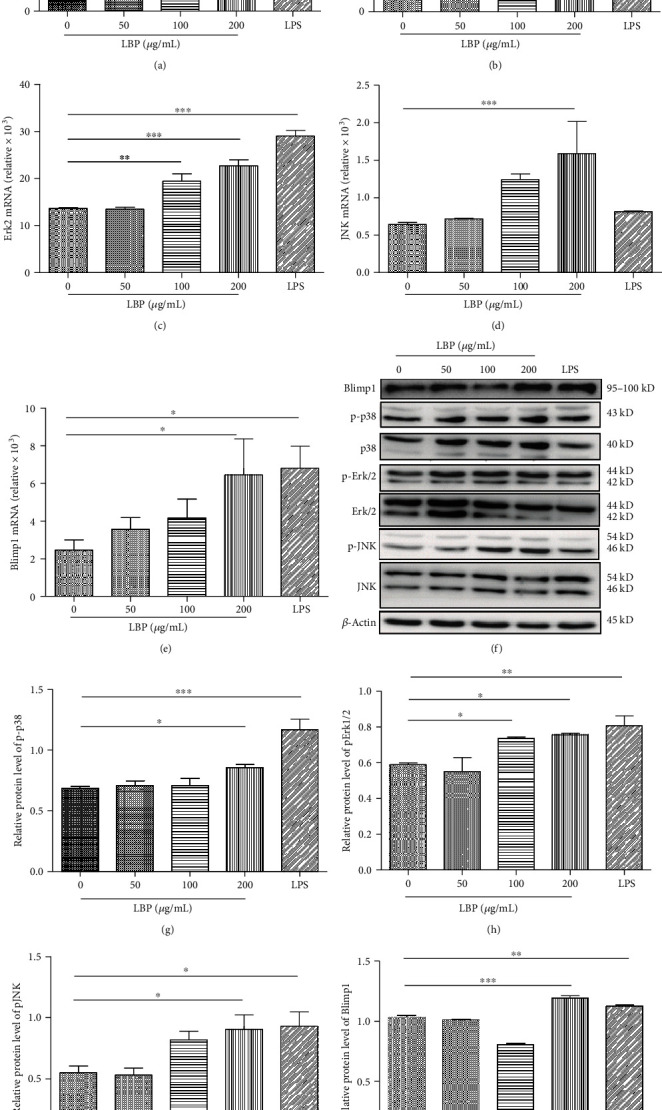
LBP increase MAPK, Blimp1 mRNA, and protein expression in DCs. DCs were stimulated for 30 min with LBP (0, 50, 100, and 200 *μ*g/mL) and LPS (100 ng/mL), respectively. After stimulation, the mRNA and protein expressions were checked as p38 (a), Erk1 (b), Erk2 (c), JNK (d), Blimp1 (e), p38 (g), Erk1/2 (h), JNK (i), and Blimp1 (j). Values are the mean and SD of 3 independent experiments. ^∗^*P* < 0.05, ^∗∗^*P* < 0.01, and ^∗∗∗^*P* < 0.001 versus control.

Considering that LBP could induce TLR4 activation on DCs, to study molecular mechanisms whether LBP affect DCs via MAPK and Blimp1 involvement and to determine MAPK and Blimp1 expression in DCs, DCs were cultured with LBP; Taqman-PCR and Western blot were performed to check the mRNA expression of p38 (Figure 3(a)), Erk1 (Figure 3(b)), Erk2 (Figure 3(c)), JNK (Figure 3(d)), Blimp1 (Figure 3(e)), phospho-p38 (Figure 3(g)), phospho-Erk1/2 (Figure 3(h)), phospho-MAPK/JNK (Figure 3(i)), and Blimp1 (Figure 3(j)). We found that mRNA and protein expression of these molecules was significantly increased, respectively, after stimulation with LBP (200 *μ*g/mL) for 30 min. These results indicate that LBP can regulate the expression of TLR4 downstream molecules p38, Erk1/2, JNK, and Blimp1 in DCs.

### 3.4. TAK 242 Reduces TLR4, Erk1/2, Blimp1 mRNA, and Protein Expression in LBP-Induced DCs

**Figure 4:**
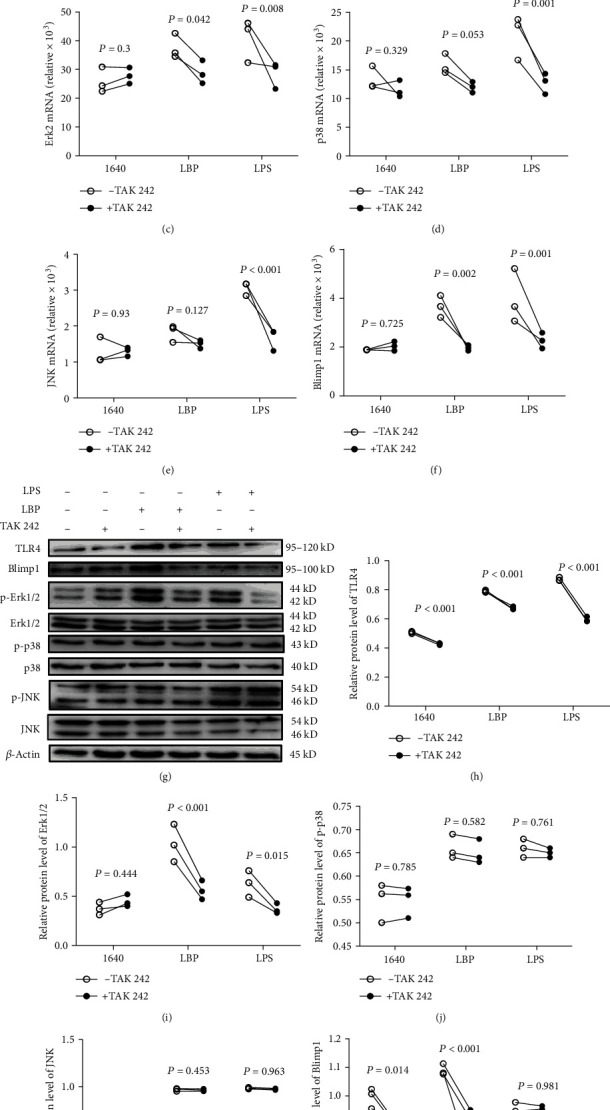
LBP regulate DCs maturation via TLR4-MAPK. DCs were pretreated with TAK 242 (10 *μ*M) for 1 h and then cocultivated with LPS (100 ng/mL) or LBP (200 *μ*g/mL) for 30 min. The mRNA and protein expression of TLR4 associated molecules was checked by Taqman-PCR and Western blot as TLR4 (a and g), Erk1 (b and h), Erk2 (c and i), p38 (d and j), JNK (e and k), and Blimp1 (f and l). Values are the mean and SD of 3 independent experiments. For the above figures, here, ^∗^*P* < 0.05, ^∗∗^*P* < 0.01, and ^∗∗∗^*P* < 0.001 were considered statistically significant different from control.

To further verify whether LBP regulate DCs via TLR4-MAPK-Blimp1, we preadministered TAK 242, to specifically inhibit the TLR4 on DCs. Results showed that compared with the RPMI-1640, TAK 242 markedly suppressed LBP-mediated upregulation of TLR4 (Figures 4(a) and 4(h)), Erk1 (Figures 4(b) and 4(i)), Erk2 (Figures 4(c) and 4(i)), and Blimp1 (Figures 4(f) and 4(l)) mRNA and protein expression. However, TAK 242 showed no effect on p38 (Figures 4(d) and 4(j)) or JNK (Figures 4(e) and 4(k)) mRNA and protein levels. These results suggested that LBP may regulate Blimp1 in DCs though TLR4-Erk1/2.

### 3.5. MAPK Inhibitors Reduce the mRNA and Protein Expression of MAPK in LBP-Induced DCs

**Figure 5:**
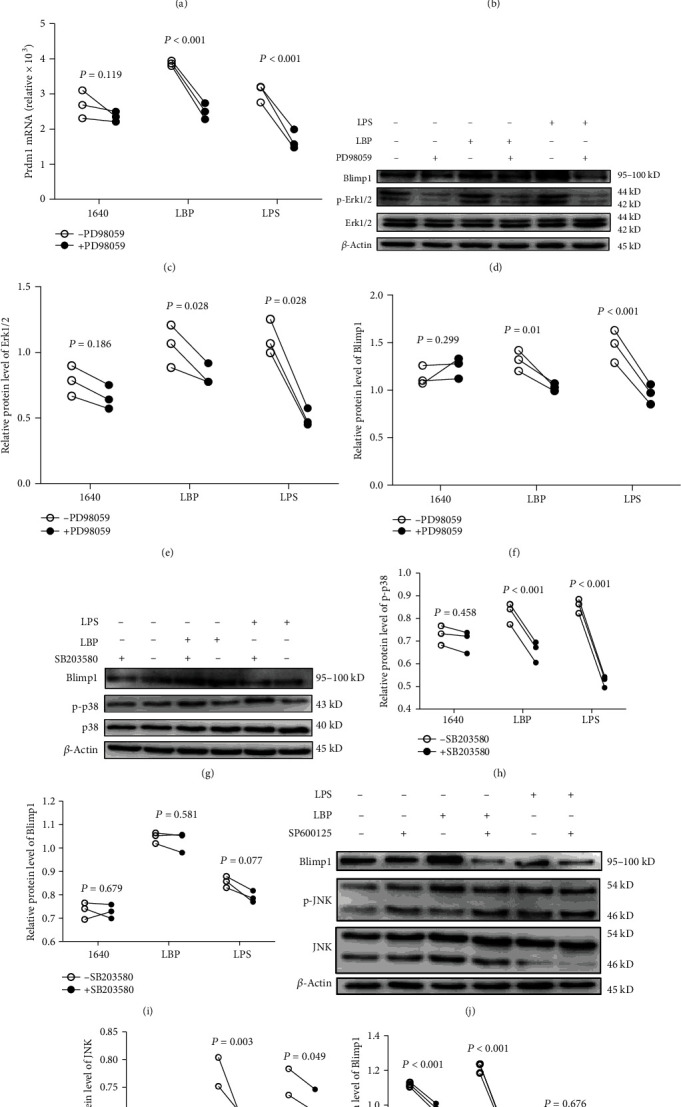
LBP regulate DCs via TLR4-MAPK-Blimp1. DCs were pretreated with 10 *μ*M PD98059, 5 *μ*M SB203580, and 50 *μ*M SP600125 for 1 h, respectively, and then treated with 100 ng/mL LPS or 200 *μ*g/mL LBP for 30 min. The mRNA and protein expression of Erk1/2 (a, b, and e), Blimp1 (c and f), p38 (h), Blimp1 (i), JNK (k), and Blimp1 (l) was analyzed by Taqman-PCR and Western blot. Values are the mean and SD of 3 independent experiments. ^∗^*P* < 0.05, ^∗∗^*P* < 0.01, and ^∗∗∗^*P* < 0.001.

To investigate the TLR4-MAPK-Blimp1 signaling pathway which LBP activates in DCs, we pretreated DCs with the Erk1/2 inhibitor PD98059, which attenuated the induction of Erk1, Erk2 mRNA, and Erk1/2 phosphorylation by LBP in DCs (Figures 5(a), 5(b), and 5(e)), indicating that Blimp1 activation by LBP occurred via canonical TLR4-Erk1/2 activation. Interestingly, when we used SB203580, which is a p38 inhibitor, to pretreat DCs, the pretreated DCs expressed less Blimp1 phosphorylation than untreated cells under either LBP or LPS stimulation (Figures 5(g)–5(i)). Correspondingly, the JNK inhibitor, SP600125, also reduces Blimp1 expression in DCs. Taken together, these results demonstrated that in the presence of LBP, TLR4-Erk1/2-Blimp1 was drastically activated, whereas p38/JNK-Blimp1 pathway may be activated through another TLR.

### 3.6. Interference Efficiency of *Prdm1* siRNA in DCs

**Figure 6:**
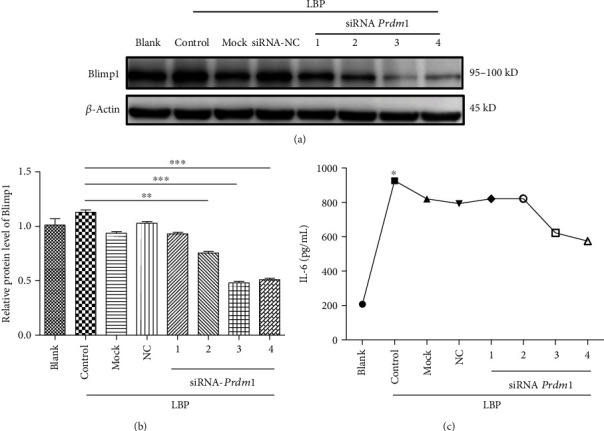
Gene silencing in LBP induced DCs using Blimp1-specific siRNA. DCs were transfected with siRNA-*Prdm1*-1, siRNA-*Prdm1*-2, siRNA-*Prdm1*-3, siRNA-*Prdm1*-4, siRNA-NC, reagent alone (mock-treated), or nontransfected cells (control). After coculture of transfected DCs with LBP for 24 h, the expression of Blimp1 was detected by Western blot (a and b), and the IL-6 concentration in the culture supernatant was detected by ELISA (c). *β*-Actin expression was treated as internal reference. Values are the mean and SD of 3 independent experiments. ^∗^*P* < 0.05, ^∗∗^*P* < 0.01, and ^∗∗∗^*P* < 0.001 versus control.

To further analyze the function of Blimp1 in DCs, we took advantage of *Prdm1* siRNA to downregulate Blimp1-specifific gene expression in DCs cultured with LBP. The expression of Blimp1 protein levels in DCs were analyzed by Western blot (Figures 6(a) and 6(b)). Following pulsing with LBP, Blimp1 protein levels in DCs were markedly increased compared with those of nonpulsed DCs (Figure 6(b)). The levels of Blimp1 protein in DCs transfected with siRNA-*Prdm1*-3 or siRNA-*Prdm1*-4 were decreased around 50% compared with DCs transfected with siRNA-NC or mock-treated DCs (Figure 6(b)). In addition, as displayed in Figure 6(c), the content of IL-6 in the cell culture supernatant added with siRNA-*Prdm1*-3 or siRNA-*Prdm1*-4 showed a downward trend. These results suggested that the *Prdm1* gene is silenced in immature DCs, the maturation of DCs will be inhibited, and the IL-6 concentration in the culture supernatant will be reduced.

### 3.7. LBP Increase TLR4, Erk1/2, and Blimp1 Expression In Vivo

**Figure 7:**
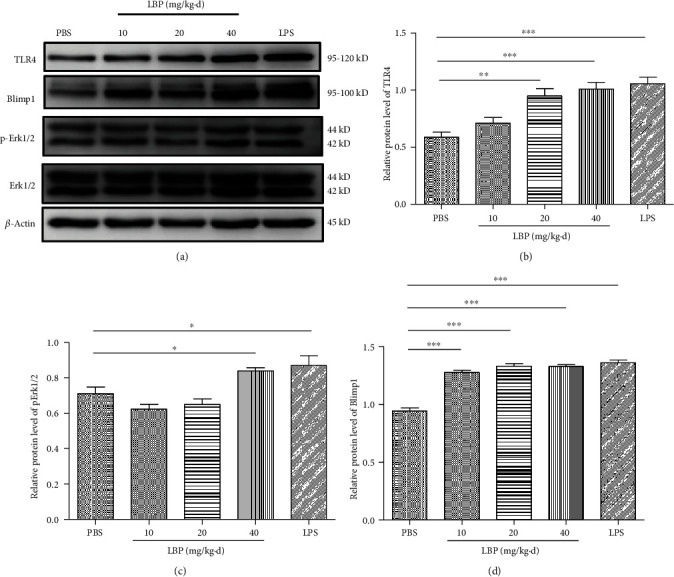
LBP induces the expression of TLR4, Erk1/2, and Blimp1 in DCs from a mouse spleen in vivo study. Both LBP (0, 10, 20, and 40 mg/kg) and LPS (1 mg/kg) were administered intraperitoneally to C57BL/6 mice in three groups. After 7 days, mouse splenic DCs were isolated and examined for TLR4 (b), Erk1/2 (c), and Blimp1 (d) expression by Western blot. Values are the mean and SD of 5 independent experiments. ^∗^*P* < 0.05, and ^∗∗^*P* < 0.01, and ^∗∗∗^*P* < 0.001 versus control.

To confirm whether LBP induces TLR4, Erk1/2, and Blimp1 expression in vivo, we injected mice intraperitoneally with LBP for 7 consecutive days. CD11c magnetic beads were used to sort DCs from the spleen cell suspension. CD11c cells were detected with flow cytometry after sorting, and the efficiency of sorting is about 70%. Then, the whole cell protein was extracted. We found the protein expression of TLR4 (Figure 7(b)), Erk1/2 (Figure 7(c)), and Blimp1 (Figure 7(d)) was significantly increased compared to the PBS group.

## 4. Discussion

DC is the body's main antigen-presenting cell and plays a key role in controlling and activating immune responses [26]. During immune response, mature DCs have stronger antigen-presenting ability than immature DCs. LPS, CpG, poly I:C, or TNF-*α* in some ways was used as an inducer of DC maturation, but their application range is limited due to toxic side effects [27]. Numerous studies have demonstrated that plant polysaccharides can also effectively induce the maturation of DCs, including Ganoderma lucidum polysaccharides, astragalus, Cordyceps sinensis, and LBP [2, 5, 28, 29].

As a well-known Chinese medicine, LBP has been considered a nourishing food content for thousands of years, and its immune regulation effects are played by its bioactive substances [30–33]. Previous studies have shown that Lycium barbarum polysaccharide liposome (LBPL) can activate immature DCs and induce DC maturation characterized by upregulation of cell surface molecules MHCII, CD80, CD86, and CD40, production of cytokines IL-12p40 and TNF-*α*, and enhancement of antigen presentation [34, 35]. In our research, we verified that LBP can induce DC maturation, which was upregulation of CD11c^+^MHCII^+^, CD11c^+^CD86^+^, and CD11c^+^CD80^+^ double-positive DCs (Figures 1(a)–1(c)) and induction of IL-6 (Figure 1(e)) and IL-4 (Figure 1(f)) production from DCs. Our results indicate that LBP (200 *μ*g/mL) can promote the expression level of DC maturation markers (Figure 1). Mature DCs were capable of secreting a variety of cytokines, mainly IL-6 and IL-4.

TLRs are classic cell surface proteins that play an important role in identifying invading pathogens while activating immune cells to produce cytokines [24]. The stimulation of TLRs leads to the activation of several transcription factors, including MAPK. Dziarski, the first scholar, reported that LPS can induce the activation of MAPK signaling pathways in macrophages, such as Erk1/2, p38, and MAPK/JNK [36]. Zhang et al. demonstrated that LBPF4-OL can significantly enhance p38 phosphorylation and inhibit JNK and Erk1/2 MAPK phosphorylation in LPS-induced macrophages [37]. However, it is not fully understood whether the MAPK signaling pathway is involved in DC activation by LBP.

Various roles of Blimp1 in B and T cell differentiation and development have been reported, but little is known about the act of Blimp1 in DCs differentiation and maturation [38]. In recent years, Chan and his collaborators found that Blimp1 is induced following maturation of DCs cultured with GM-CSF [39]. However, the molecular mechanism by which Blimp1 activates DCs still remains elusive. Therefore, we are interested to explore whether Blimp1 is involved in the downstream signaling molecules of TLR4-Erk1/2 in LBP-regulated DCs in this study.

Our studies showed that 200 *μ*g/mL of LBP significantly induced the mRNA and phosphorylated protein expression of TLR4, p38, Erk1/2, JNK, and Blimp1 in DCs compared with RPMI-1640 stimulation. After treatment of DCs with the TLR4 inhibitor (TAK 242), mRNA and protein expression of LBP-induced TLR4, Erk1/2, and Blimp1 was significantly downregulated. At the same time, we used the Erk1/2 inhibitor (PD98059) to treat DCs; we found that the mRNA and protein expression of LBP-induced Erk1/2 and Blimp1 was inhibited. More interestingly, TAK 242 had no significant effect on the expression of p38 and JNK proteins; however, the level of Blimp1 protein was decreased after the treatment of SB203580 and SP98059.

*PRDM1*, which encodes Blimp1, plays an indispensable role in the differentiation and development of a variety of cells [17]. After silencing *Prdm1* in DCs, we were surprised to find that there was a downward trend in IL-6 secretion by DCs, considering that the maturation of DCs might be impaired due to knockdown of Blimp1, and thus, IL-6 secretion was reduced. In summary, LBP regulated the maturation of DCs via the TLR4-Erk1/2-Blimp1-dependent pathway and promoted IL-6 production (Supplementary Graphical Abstract).

## 5. Conclusion

We verified that LBP were capable of inducing the maturation of DCs by increasing MHCII, CD80, and CD86 expression. Additionally, we found that LBP could enhance the mRNA and protein expression of TLR4, p38, Erk1/2, JNK, and Blimp1 during DC maturation. More importantly, we found that LBP can promote maturity of murine DCs through the TLR4-Erk1/2-Blimp1 signaling pathway.

## Data Availability

The data used to support the findings of this study are available from the corresponding author upon reasonable request.

## Supplementary Materials

Supplementary Materials LBP mediates the maturation of murine DCs through the TLR4 pathway. LBP regulated the maturation of DCs via the TLR4-Erk1/2-Blimp1-dependent pathway and promoted IL-6 production. These signaling pathways may provide a novel evidence for understanding the molecular mechanisms of LBP on activating murine DCs.
