# Supplementary material for: Lycium barbarum Polysaccharides Promote Maturity of Murine Dendritic Cells through Toll-Like Receptor 4-Erk1/2-Blimp1 Signaling Pathway
Source: J Immunol Res. 2020 Dec 1;2020:1751793. doi: 10.1155/2020/1751793 (PMC7725586; doi:10.1155/2020/1751793)
Supplement: Supplementary Materials — LBP mediates the maturation of murine DCs through the TLR4 pathway. LBP regulated the maturation of DCs via the TLR4-Erk1/2-Blimp1-dependent pathway and promoted IL-6 production. These signaling pathways may provide a novel evidence for understanding the molecular mechanisms of LBP on activating murine DCs. [file 1751793.f1.docx]

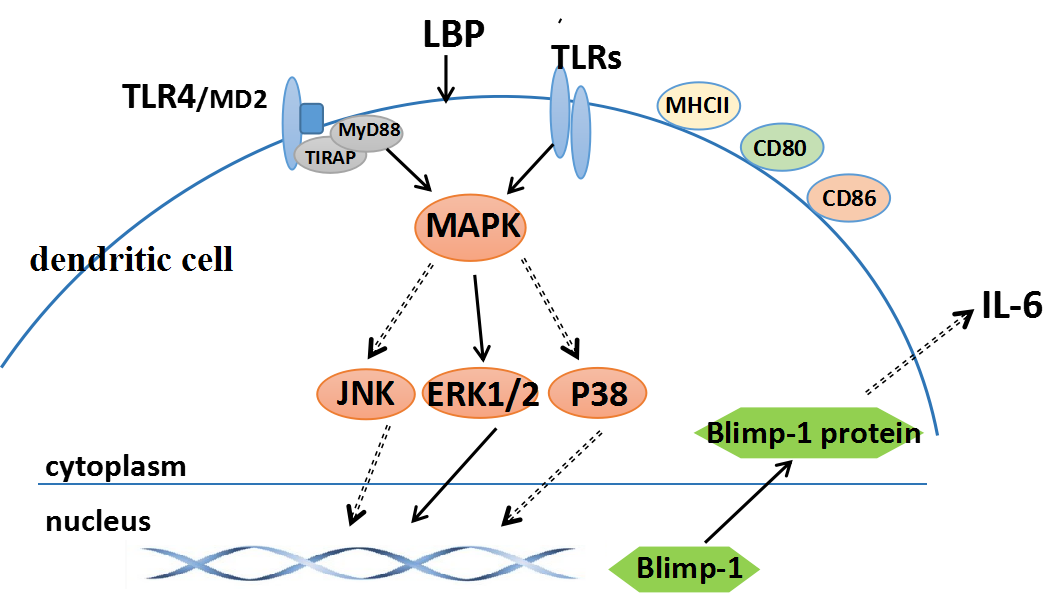


**Graphical Abstract**.

LBP mediates the maturation of murine DCs through the TLR4 pathway. LBP promote maturity of murine DCs through TLR4-Erk1/2-Blimp1 signaling pathway which may provide a novel evidence for understanding the molecular mechanisms of LBP on activating murine DCs. (The full line arrows signify promotion; the dotted arrows signify a rising trend but no statistical difference.)
